# Therapeutic role of Wuda granule in gastrointestinal motility disorder through promoting gastrointestinal motility and decreasing inflammatory level

**DOI:** 10.3389/fphar.2023.1237686

**Published:** 2023-08-21

**Authors:** Zhi Jiang, Qiuping Zou, Qicheng Chen, Junhong Zhang, Hailin Tang, Jingbao Chen, You Qin, Liming Yang, Zhiqiang Chen, Lixing Cao

**Affiliations:** ^1^ Department of Perioperative Research Centre of Chinese Medicine, The Second Affiliated Hospital of Guangzhou University of Chinese Medicine, Guangzhou, China; ^2^ Emergency Department, Dongguan People’s Hospital, Dongguan, China; ^3^ Department of Research Public Service Center, The Second Affiliated Hospital of Guangzhou University of Chinese Medicine, Guangzhou, China; ^4^ State Key Laboratory of Oncology in South China, Sun Yat-Sen University Cancer Center, Guangzhou, China; ^5^ Department of Minimally Invasive Surgery, The Second Affiliated Hospital of Guangzhou University of Chinese Medicine, Guangzhou, China

**Keywords:** gastrointestinal motility disorders, network analysis, Wuda granule, molecule docking, Chinese herb medicine

## Abstract

**Introduction:** Previous studies indicated that Wuda Granule (WDG) has been applied in the treatment of gastrointestinal motility disorder (GMD), but the effect and underlying mechanisms is yet to be elucidated. This study aimed to explore the mechanism and pharmacological effect of WDG for GMD via network analysis, verification of animal experiments and clinical experiments.

**Methods:** The chemical components of WDG were identified from the Traditional Chinese Medicine Systems Pharmacology Database (TCMSP, http://lsp.nwu.edu.cn/index.php), and the Encyclopedia of Traditional Chinese Medicine (ETCM, http://www.tcmip.cn/ETCM/index.php/Home/Index/) according to oral bioavailability (OB) ≥ 20% and drug-likeness (DL) ≥ 0.10. The targets of WDG compounds were retrieved from the Swiss Target Prediction database (http://www.swisstargetprediction.ch/) and targets related to GMD were retrieved from GeneCards database (https://www.genecards.org/). Network analysis were performed to screen the key active compounds of WDG and its hub targets. Then the pharmacological effect of WDG were verified via vivo experiments in rats and clinical experiments.

**Results:** The results showed that 117 effective active compounds of WDG were screened and 494 targets of WDG compounds targeting GMD were selected. These targets were involved in the biological process of inflammatory regulation and the regulation of gastrointestinal motility. The mechanism was mainly involved in the regulation of PI3K-Akt signaling pathway and Rap1 signaling pathway. In addition, molecular docking analysis suggested that eight key active compounds of WDG may be mainly responsible for the effect of WDG on GMD by targeting HARS, AKT, and PIK3CA, respectively. Animal experiments and clinical trials both suggested that WDG could exert therapeutical effect on GMD via inhibiting inflammation and promoting gastrointestinal motility, it could also improve digestive function of patients with laparoscopic colorectal cancer after surgery.

**Conclusion:** This study was the first to demonstrate that WDG improved GMD mainly via inhibiting inflammatory level and promoting gastrointestinal motility, providing new insights for the understanding of WDG for GMD, inspiration for future research and reference for clinical strategy in terms of the treatment of GMD.

## 1 Introduction

Gastrointestinal motility disorder (GMD), affecting millions of patients worldwide (Madl and Madl, 2018), is an important mechanism of functional gastrointestinal diseases and mainly presents as debilitating symptoms, delayed gastric emptying, and unexpected severe gastric symptoms (Suresh et al., 2021). Especially for the majority of patients undergoing gastrointestinal surgery, GMD may directly lead to bowel failure (Foong et al., 2020). The incidence of GMD is increasing with aging society (Singh et al., 2022), and is associated with the functional degradation or loss of neurons in the enteric nervous system as well as the intrinsic innervation of the bowel, which is a major contributing factor to gastrointestinal motility. In addition, GMD can be secondary to other neurological, autoimmune, or metabolic diseases, paraneoplastic syndromes, endocrine disorders, and infectious diseases. However, there is a lack of approved and efficacious drugs for GMD (Docsa et al., 2022), which highlights the need for the development and deployment of new or alternative drugs with potential effectiveness.

Traditional Chinese medicine (TCM), with thousands of years of practice and experience, is a major source of new drugs. It is mainly comprised of natural products, and has been used in the prevention and treatment of human diseases (Yang et al., 2013). Wuda granule (WDG), a traditional Chinese patent medicine, is consists of the following five herbal medicines: *Arecae Semen* [Arecaceae; Areca catechu L.], *Panax ginseng* [Araliaceae; Ginseng Radix et Rhizoma], *Lindera aggregata* (Sims) *Kosterm* [Lauraceae; Linderae Radix], *Persicae Semen* [Rosaceae; Prunus persica (L.) Batsch] and *Fructus amomi* [Zingiberaceae; Amomum villosum Lour.] (Jiang et al., 2020). The major components of WDG are ginsenoside Rc, ginsenoside Rd, ginsenoside Rg1, quercetin, quercitrin, isoquercitrin, laetrile, norisoboldine, linderane, and *arecoline*, etc. (Wang et al., 2020). Additionally, it mainly comprises four alkaloids, including arecoline, norisoboldine, and boldine (Wang et al., 2020). It has been proved that WDG treatment for GMD can improve gastrointestinal function in practice (Jiang et al., 2017; Zeng et al., 2022), but the underlying mechanism still lacks further verification and remains to be elucidated.

Network analysis is an effective method for comprehensively analyzing complex networks using high-throughput analyses and computerized calculations (Wang et al., 2021). The holistic philosophy of TCM is consistent with the key ideas of emerging network analysis and the requirements for treating complex diseases (Li and Zhang, 2013). This approach can be used to explore interactions between various compounds, diseases, genes, and proteins (Lai et al., 2020). Recently, an increasing number of studies have used network analysis to reveal the mechanisms and effects of TCM in the treatment of diseases. The aim of this study was to explore the possible biological function of WDG and its mechanism of action in GMD via the network analysis and virtual molecular docking analyses, the findings of which were further verified via *in vivo* experiments and clinical trials.

## 2 Materials and methods

### 2.1 Network analysis

#### 2.1.1 Identification of WDG compounds and their targets

The chemical components of WDG were identified from the Traditional Chinese Medicine Systems Pharmacology Database (TCMSP, http://lsp.nwu.edu.cn/index.php), and the Encyclopedia of Traditional Chinese Medicine (ETCM, http://www.tcmip.cn/ETCM/index.php/Home/Index/). Candidate compounds with oral bioavailability (OB) ≥ 20% and drug-likeness (DL) ≥ 0.10 of WDG were screened. The chemical structure formulae of the screened compounds was obtained from the PubChem database, and the targets of WDG compounds were retrieved from the Swiss Target Prediction database (http://www.swisstargetprediction.ch/).

#### 2.1.2 Identification of targets related to GMD and screening by venn analysis

The therapeutic targets of GMD were retrieved from GeneCards database (https://www. genecards. org/), which is a comprehensive multifunctional database containing the therapeutic targets of all known human diseases. The query keywords used were “gastrointestinal mobility disorder”, “gastrointestinal motility, or “gastrointestinal peristalsis”. To exclude the interference of targets with low correlation with GMD, only targets with a relevance score greater than or equal to the median value of all the targets were included. Intersection targets between GMD proteins and WDG targets were screened by Venn analysis, and were considered as targets of WDG compounds with potential pharmacological therapeutic effects on GMD.

#### 2.1.3 Analysis of protein-protein interaction network

The protein-protein interaction (PPI) of intersection targets was retrieved from the STRING database (https://string-db.org/), which was used to search and predict protein-protein interactions, and identify core regulatory genes in the PPI network. Core targets with functional interactions were screened according to the degree value in the network after network topology analysis.

#### 2.1.4 Construction of networks

The herbal-compounds-targets (HCT) and PPI networks were constructed, visualized, and analyzed using the open-source Cytoscape software (Version 3.7.1). Network topology analysis was conducted to determine important compounds and core targets of WDG in the HCT and PPI networks, respectively, which calculated the value of the degree between the nodes, thereby suggesting the interaction strength between the compounds, targets, or herbs.

#### 2.1.5 Gene ontology enrichment and kyoto encyclopedia of gene and genome pathway enrichment

To elucidate the potential mechanisms of action of WDG in the treatment of GMD, targets with twice more than the median value of degree in the PPI network were selected for gene ontology (GO) enrichment and Kyoto Encyclopedia of Gene and Genome (KEGG) pathway enrichment analyses using the DAVID database (DAVID 6.8, https://david.ncifcrf.gov/). An adjusted *p* value less than 0.05 indicated that the GO terms and the pathway were significantly associated with WDG. Finally, the results of GO and KEGG enrichment were visualized via the bubble plot using the “ggplot2” package of R software 4.2.0.

#### 2.1.6 Verification of the key active compounds and key targets of WDG by molecular docking

In order to determine whether the screened active compounds of WDG could bind to the core target, thus providing evidence for our predicted results on the active compounds of WDG and their potential therapeutical targets, we conducted molecular docking analysis to verify the results. The structures of the core targets were obtained from the PDB database (https://www.rcsb.org/) and * SDF format files of the screened active WDG compounds were downloaded from the PubChem database (https://pubchem.ncbi.nlm.nih.gov/). The bioactive compounds of WDG (ligands) were examined for their putative antibacterial activities against key targets to determine efflux protein-ligand interactions through molecular docking analysis using the Schrodinger software suite.

### 2.2 Verification by *in vivo* experiments

Recent studies have demonstrated a link between the immune inflammatory response and the occurrence and development of gastrointestinal dysfunction. Our network analysis also indicated that WDG might exert a therapeutic effect on GMD through its anti-inflammatory effects. Rat models were chosen to validate the anti-inflammatory effect of WDG on GMD because the immune system is activated in GMD rat models and large number of inflammatory factors are increasingly released during the stress response (Patil et al., 2019; Accarie and Vanuytsel, 2020).

#### 2.2.1 Animals

Forty specific pathogen free grade SD rats (weighing 150–200 g each) were purchased from the Guangdong Medical Laboratory Animal Center (animal quality certificate: 44007200051802). Ethics approval for the study protocol was obtained from the Institutional Animal Care and Ethics Committee of Guangdong Provincial Hospital of Chinese Medicine (certificate number: 2017041).

#### 2.2.2 Experimental design

Forty rats were randomly divided into four groups: control group (normal rat + 0.9% NaCl), Postoperative ileus (POI) group (POI rat + 0.9% NaCl), WDG group (POI rat + Wuda granule 500 mg/kg), and prucalopride group (POI rat + prucalopride 2 mg/kg). Rats in the control and POI groups were administered 100 μ L of 0.9% NaCl solution, whereas those in the WDG and prucalopride groups were administered the same volume of 500 mg/kg WDG and 2 mg/kg prucalopride, respectively, at 6, 12, 18, and 24 h after the surgery.

#### 2.2.3 Surgical procedures

Before POI induction, the rats were denied food for 12 h and kept water-free for 6 h. After anesthesia, the rats were placed in the supine position and immobilized using tape following which their hair was shaved, the abdomen was sterilized with iodine solution, and the abdominal cavity was opened with surgical scissors. Thereafter, the small intestine to the cecum region was placed on sterilized gauze, and the entire small intestine was massaged three times using a sterile cotton swab (Kalff et al., 1998). Finally, the abdomen was sutured, and the duration of the entire operation did not exceed 10 min. Rats were gavaged with the corresponding drugs at the same time points (6, 12, 18, or 24 h) postoperatively. Specimens were collected 24 h after surgery, including the mesenteric tissue and blood from the abdominal aorta.

#### 2.2.4 Detection of serum VIP and cytokines

Blood collected from each group was placed in a centrifuge tube which was incubated at 4°C for 3 h and then centrifuged at 3000 rpm for 15 min. The upper serum was collected and used for Enzyme Linked Immunosorbent Assay (ELISA) of VIP and IL-10 according to the manufacturer’s instructions. Optical density was measured at 450 nm using a SpectraMax M5 autoreader.

#### 2.2.5 Preparation of gastrointestinal smooth muscle specimen

Half an hour before the preparation of the rat gastrointestinal smooth muscle specimen, cold water was added to the external circulation bath of the HV-4 isothermal tissue-organ perfusion system to maintain the temperature at 37°C. Thereafter, 10 mL of Krebs solution was injected into the bath, and a mixture of 95% O_2_ and 5% CO_2_ was continuously injected into the bath via the gas supply device. After 4 h of reperfusion, the rats were anesthetized by an intraperitoneal injection of 7% chloral hydrate. The abdomen was opened and the gastric antrum, duodenum, and jejunum were rinsed with Krebs solution at room temperature. Thereafter, the tissues were fixed to the bottom of a glass dish using a silicone plate, and the chassis was filled with Krebs solution. Finally, the mucosal layer was removed using tweezers. Based on the direction of the longitudinal muscle fiber, 8 mm × 2 mm longitudinal smooth muscle strips were cut using a blade following which each longitudinal muscle strip was suspended in the tissue chamber. One end of the strip was fixed to a hook at the bottom of the chamber and the other was connected to an external isometric force transducer. The strips were subjected to an initial tension of 1 g and were balanced for 60 min. The Krebs solution was replaced every 15 min during the balancing process, and the spontaneous contraction frequency and area under the curve were calculated. After the muscle strips contracted smoothly, 100 μL of acetylcholine (5–10 mol/L) was added to the muscle groove. The amplitude index, contraction tension, and contraction frequency of the muscle strips before and after the administration were recorded by Protowin.

### 2.3 Clinical experiment

Gastrointestinal motility disorder (GMD) is a common gastrointestinal disease that severely affects the patient’s life and physical wellbeing (Raubinger et al., 2022). It is the main complication or symptom of irritable bowel syndrome and other functional gastrointestinal disorders, particularly in patients undergoing gastrointestinal surgery (Wang et al., 2019). Thus in order to validate the effect of WDG and understand its potential mechanism, we recruited patients with colon or rectal cancer who had undergone colorectal cancer surgery. According to the results of the network analysis, WDG may exert a therapeutic effect on GMD via its anti-inflammatory effects and by promoting gastrointestinal motility. In practice, GMD is a common complication among patients with colon or rectal cancer who have undergone colorectal cancer surgery.

#### 2.3.1 Ethical statement

Informed consent was obtained from all the participants, and the study protocol was approved by the Ethics Committee of Guangdong Provincial Hospital of Chinese Medicine (approval number: B2016-046-01). This study was registered with the Chinese Clinical Trial Registry (registration number: ChiCTR-IPR-16008599). All participants were informed about the trial content and signed an informed consent form before the trial.

#### 2.3.2 Participants in the experiment

Patients were recruited according to the following criteria:1) diagnosed with distinct histopathological evidence of colon or rectal cancer; 2) underwent colorectal cancer surgery at the Guangdong Provincial Hospital of TCM (The Second Affiliated Hospital of Guangzhou University of TCM); 3) the duration of the surgical procedure was within 2–4  h; 4) the duration under anesthesia was within 2.5–4.5  h; 5) did not experience serious complications, infections, adverse events, or secondary surgery during treatment; and 6) levels of serum albumin >27 g/L and prealbumin > 0.14 g/L.

Forty patients with who had undergone laparoscopic colorectal cancer surgery were included in this study. Of these, 26 patients (14 of the WDG and 12 of the placebo group) completed the entire experiment, while 14 dropped out. Of the 14 who dropped out, eight patients were of the placebo group and six were of the WDG group. Of the eight patients of the placebo group who dropped out, three patients withdrew because the duration of surgery exceeded the time stipulated in the inclusion criteria, three patients withdrew because they could not tolerate the piezometric tube after the surgery, and two patients accidentally pulled out the piezometric tube due to pain and irritation at the port of surgery. Of the six patients of the WDG group who dropped out, three patients withdrew because the duration of surgery for these exceeded the time stipulated in the inclusion criteria, and three patients were unwilling to continue participating in the trial after surgery and voluntarily withdrew. All 26 patients who completed the entire experiment received the same fundamental treatment. The WDG treatment group received fundamental treatment plus WDG, whereas the placebo group received fundamental treatment only.

#### 2.3.3 Gastrointestinal pressure measurement

Gastrointestinal pressure was measured using a gastrointestinal motility instrument (MMS, Netherlands) comprising a 24-channel high-resolution pressure measurement system. A gastrointestinal piezometer tube was sterilized and placed in the abdomen of the patient a day before surgery. Briefly, after topical anesthesia was administered to the nasal cavity, the tube was slowly inserted from one side of the nasal cavity. After successful placement, the tube was fixed. Monitoring of the gastrointestinal motility of the patient was initiated 2–3 h before the surgery. After surgery, gastrointestinal motility was monitored for 2–3 h on an empty stomach for 1–3 days. To measure gastrointestinal pressure, the patient was intubated, and the tube was located at the proximal end of the jejunum using radiography. According to the characteristics of the contraction wave, the pressure wave of the gastric antrum was wide with a high amplitude and that of the duodenum was narrow and sharp. The frequency of sinus contractions was approximately 3 contractions/min, and that of the duodenum and jejunum was 8–12 times/min.

#### 2.3.4 Measurement of postoperative related indicators

Migrating motor complex (MMC) parameters of the gastric antrum, duodenum, and jejunum, including the duration of phases I and II, the contraction amplitude and frequency of phase III, were mainly determined using the measurements of high-resolution gastrointestinal pressure. The MMC parameters were also related to the motor index (MI) of the jejunum, duration, MMCII phase amplitude, and MI. Clinical evaluation indicators of postoperative gastrointestinal motility recovery included the first postoperative gas level, duration of defecation, duration of postoperative liquid and semi-liquid diet recovery, duration of hospitalization, and hospitalization expenses.

#### 2.3.5 Determination of cytokine level and endotoxin in serum and colon tissue

Blood samples were collected from the patients at 8:00 a.m. on the first, second, third, and seventh postoperative days. Each sample was centrifuged at 3000 rpm for 10 min at 4°C, following which 1.5 mL of the supernatant was transferred to a fresh centrifuge tube and stored at −80°C. Levels of IL-4, IL-6, TNF-α and endotoxin in serum and colon tissue were measured by ELISA kits according to the manufacturer’s instructions.

### 2.4 Statistical analyses

All experimental data was presented as the mean ± standard deviation and was analyzed by one-way ANOVA test followed by Bonferroni test for multiple comparisons using GraphPad Prism software. Student’s t-tests and Mann–Whitney U tests was used to assess statistical significance between two groups (version 8.0, GraphPad Software Inc., United States). Two-factor ANOVA was used to compare different time points. A value of *p* < 0.05 was considered statistically significant.

## 3 Results

### 3.1 WDG may exert therapeutical effect on GMD via multi-compounds

**FIGURE 1:**
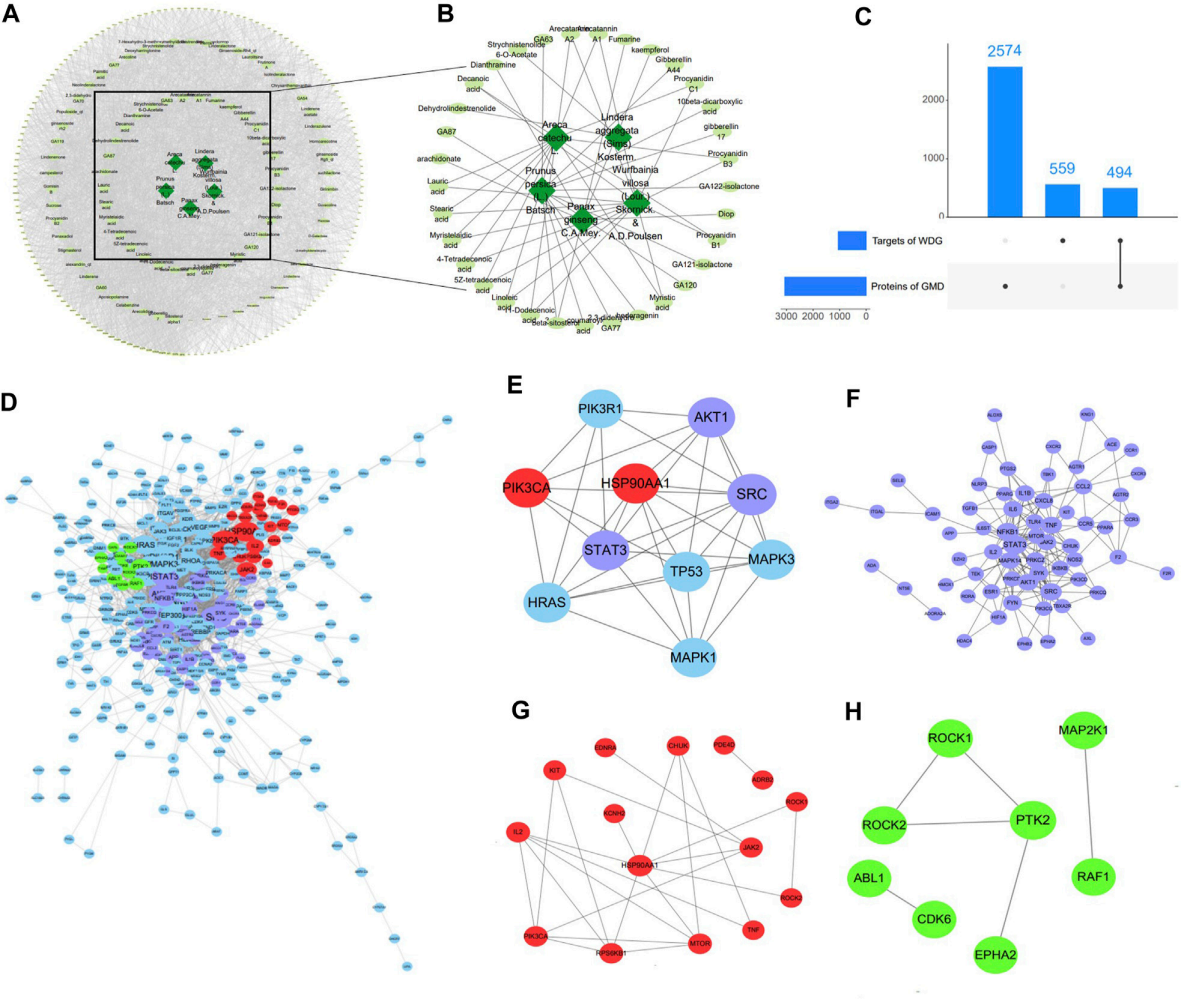
WDG may exert therapeutical effect on GMD via multi-compounds and multi-targets. (**(A)** Herbs-Compounds-Targets network of WDG; **(B)** Screened active compounds of WDG; **(C)** Intersection targets between proteins of GMD and targets of WDG compounds; **(D)** PPI networks of intersection targets; Green: targets related to gastrointestinal motility; Red: targets related to regulation of gastrointestinal smooth muscle contraction; Purple: targets related to regulation of inflammation; **(E)** 10 hub targets with node degrees 5.64 fold greater than the average node degree; **(F)** Network of targets related to regulation of inflammation; **(G)** Network of targets related to regulation of gastrointestinal smooth muscle contraction; **(H)** Network of targets related to regulation of gastrointestinal motility).

After removing duplication, 117 compounds of WDG were obtained according to OB ≥ 20% and DL ≥ 0.10. A total of 1053 predicted targets of the 117 WDG compounds were then identified. As shown in Figure 1A, the Herbs-Compounds-Targets network consisted of 255 nodes and 4043 edges, the average degree node of the common compounds was 48.23, and eight hub compounds, whose node degrees were 2.11fold greater than the average degree in this network, were identified. The respective nodes for these were 107 for beta-sitosterol (PubChem CID:222284), 102 for 5Z-tetradecenoic acid (PubChem CID:5312400), 102 for 4-tetradecenoic acid (PubChem CID:5282739), 102 for myristelaidic acid (PubChem CID:5312402), 102 for stearic acid (PubChem CID:5281), 102 for 11-dodecenoic acid (PubChem CID:125207), 102 for lauric acid (PubChem CID:3893), and 102 for linoleic acid (PubChem CID:5280450)), suggesting that these eight hub compounds might represent the main active compounds of WDG, and further suggesting that WDG may exert a therapeutic effect on GMD via multi-compounds (Figure 1B).

### 3.2 WDG may exert therapeutical effect on GMD via multi-targets

A total of 3073 GMD targets were screened in the GeneCards database after removing duplication. Subsequently, 494 intersection targets were selected via Venn analysis between the targets of the WDG compounds and GMD targets (Figure 1C). The intersection targets were regarded as targets of the WDG compounds targeting GMD. The PPI network of intersection targets was retrieved with a minimum required interaction score > 0.9, as shown in Figure 1D, which contained 396 nodes and 2038 edges with an average degree of 10.29. A total of 10 hub targets were identified (Figure 1D) with degrees greater than 58 and 5.64fold greater than the average node degree in this network (82 for SRC, 67 for PIK3R1, 67 for TP53, 66 for HSP90AA1, 64 for STAT3, 61 for PIK3CA, 60 for MAPK1, 60 for MAPK3, 59 for HRAS, and 58 for AKT1) (Figure 1E). Multi-targets related to the regulation of inflammation, gastrointestinal smooth muscle contraction, and gastrointestinal motility were clustered in the PPI network (Figures 1F–H). These results indicated that WDG may serve as an agent with multi-compounds and exerts therapeutic effects on GMD via multi-targets.

### 3.3 WDG may exert therapeutic effect on GMD via the regulation of inflammation and gastrointestinal motility

**FIGURE 2:**
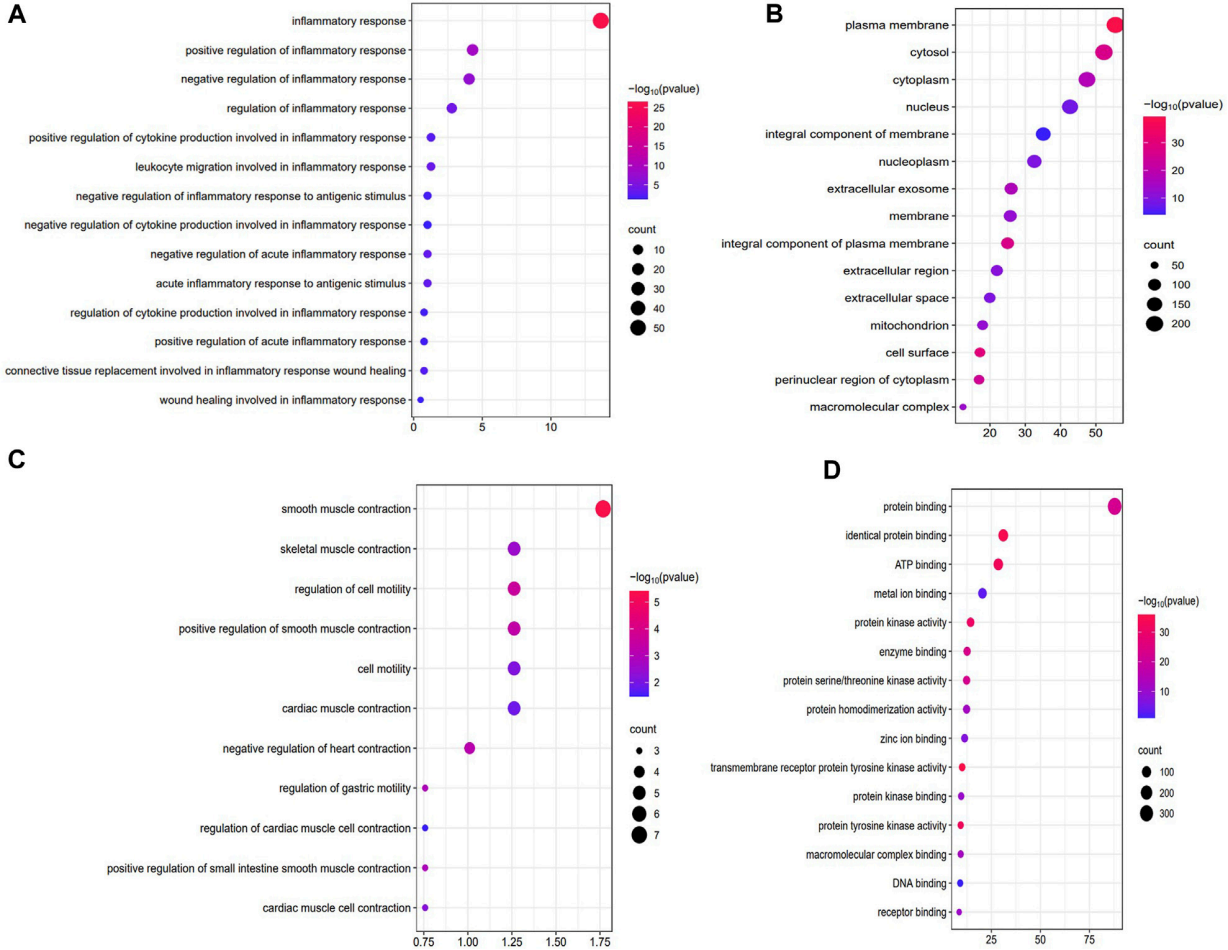
WDG may exert therapeutic effect on GMD via the regulation of inflammation and gastrointestinal motility in the GO enrichment of Biological processes (BP), Cellular component (CC), Molecular function (MF). (**(A)** Regulation of inflammation of biological processes (BP); **(B)** Regulation of inflammation of biological processes (BP); **(C)** Results of cellular component (CC); **(D)** Results of molecular function (MF)).

Figure 2 showed the results of the GO enrichment analysis of targets in the PPI network. Among the biological processes, the intersection targets were mainly enriched in the inflammatory process, including inflammatory response, regulation of inflammatory response, leukocyte migration involved in inflammatory response, regulation of acute inflammatory response, acute inflammatory response to antigenic stimulus, regulation of cytokine production involved in inflammatory response, regulation of inflammatory response to antigenic stimulus, and regulation of cytokine production involved in inflammatory response (Figure 2A). The targets were also enriched in the regulation of gastrointestinal motility, including smooth muscle contraction, positive regulation of smooth muscle contraction, positive regulation of small intestine smooth muscle contraction, skeletal muscle contraction, regulation of cell motility, and regulation of gastric motility (Figure 2B). In addition, targets enriched in molecular function mainly included protein binding, ATP binding, protein kinase activity, enzyme binding, DNA binding, and receptor binding (Figure 2C). Further, targets enriched in cellular component, mainly included the plasma membrane, cytosol, nucleus, extracellular region, and mitochondria (Figure 2C). These results suggested that WDG may regulate the inflammation process and gastrointestinal motility via protein binding, ATP binding, and protein kinase activity in the plasma membrane, cytosol, nucleus, extracellular region, and mitochondria, etc.

### 3.4 WDG may exert therapeutic effect on GMD via the regulation of multi-pathways

**FIGURE 3:**
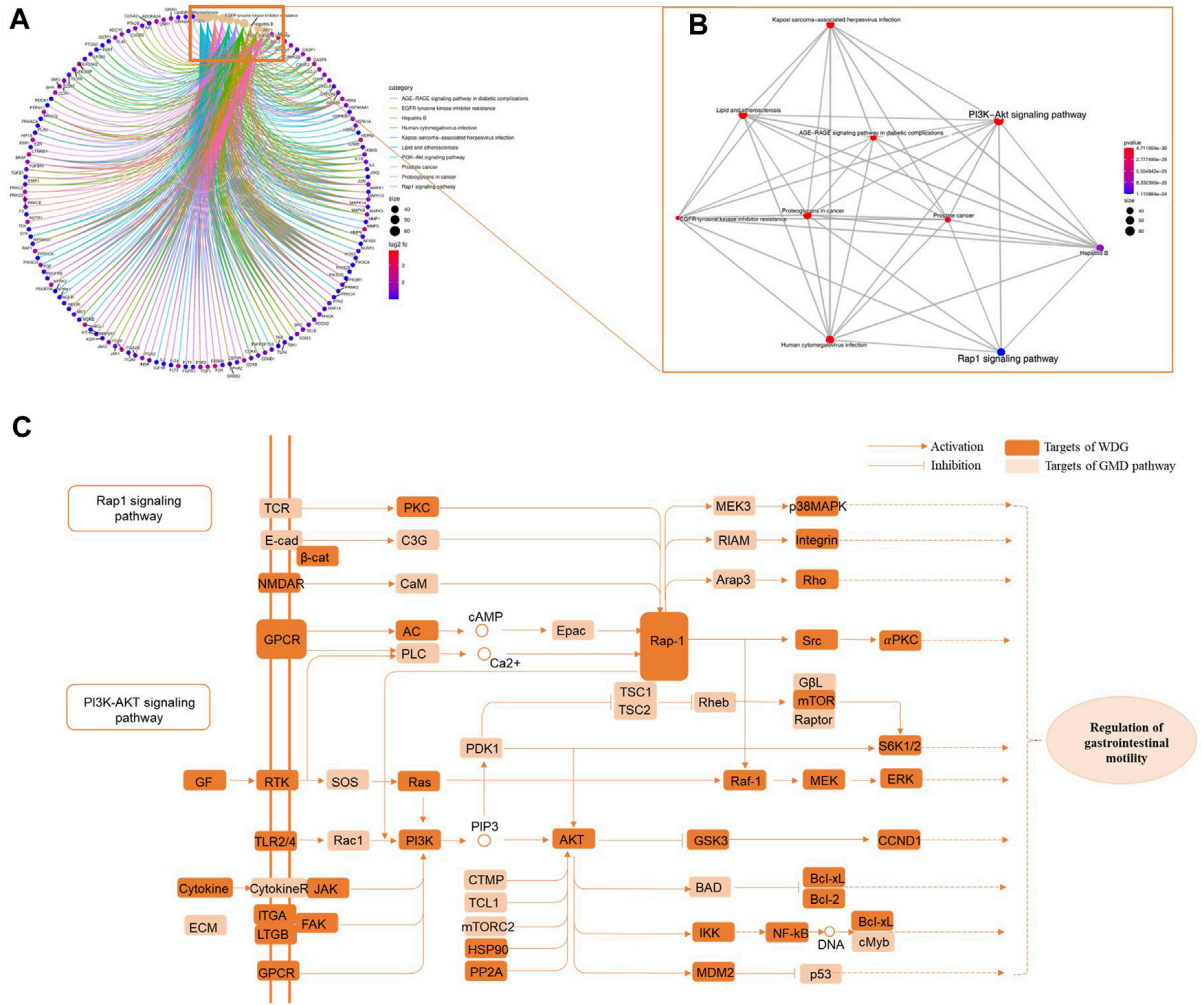
WDG may exert potential therapeutical effect on GMD via regulating multi-pathways. (**(A)** Results of KEGG pathway enrichment; **(B)** gene-concept network analysis on KEGG enrichment; and **(C)** “GMD-pathway” network of pathway crosstalk between PI3K-Akt signaling pathway and Rap1 signaling pathway).

The results of KEGG pathway enrichment showed that WDG was mainly involved in the regulation of the PI3K-Akt and Rap1 signaling pathways (Figures 3A, B), after excluding specific pathways related to specific diseases, such as lipid and atherosclerosis, the AGE-RAGE signaling pathway in diabetic complications, prostate cancer, and Hepatitis B. Figure 3C showed that intersection of pathway crosstalk between PI3K-Akt signaling pathway and Rap1 signaling pathway were integrated into an “GMD-pathway” network, which indicated that WDG may exert therapeutic effect on GMD via the regulation of multi-pathways.

### 3.5 WDG decreased inflammation in POI rats

**FIGURE 4:**
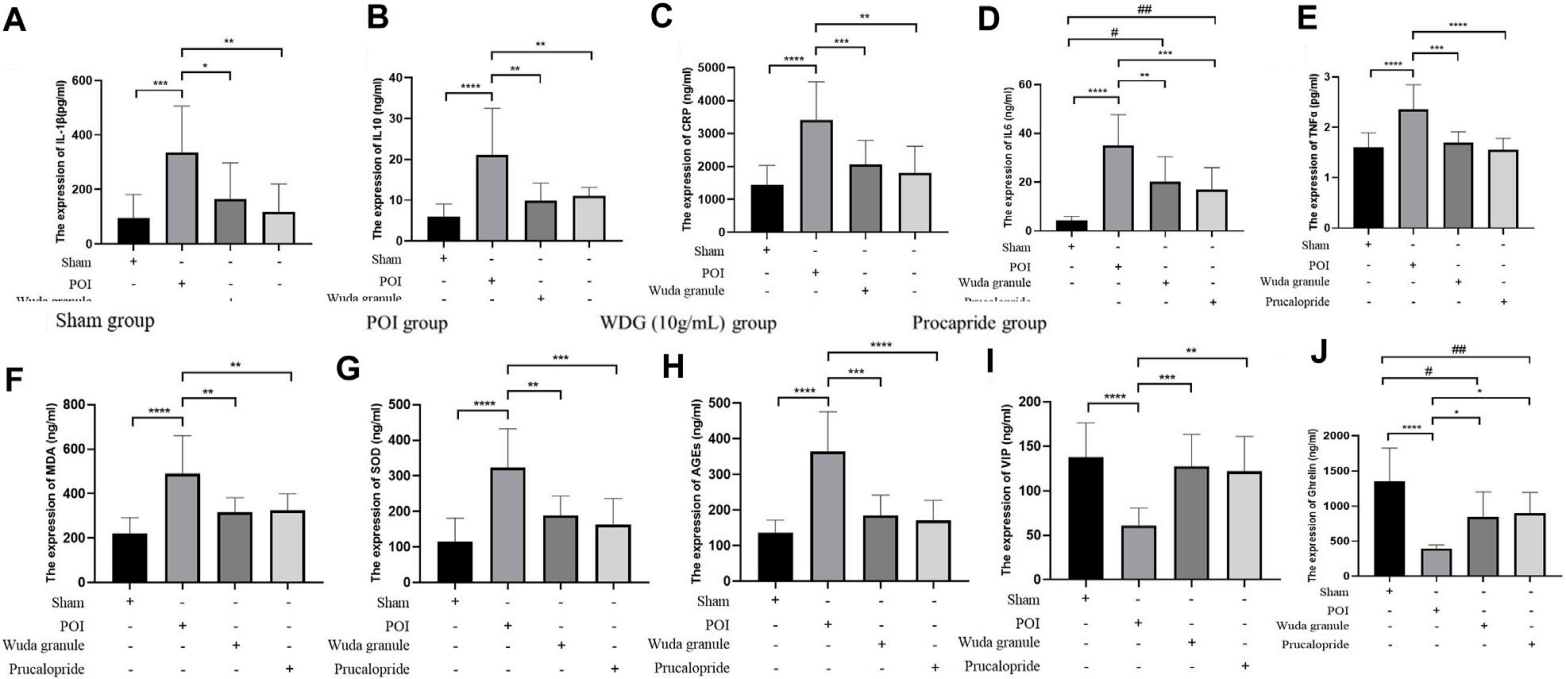
WDG decreased inflammation expression and improved gastrointestinal motility in Rats. (**(A)** The expression level of IL-1β; **(B)** The expression level of IL-10; **(C)** The expression level of CRP; **(D)** The expression level of IL-6; **(E)** The expression level of TNF α ; **(F)** The expression level of MDA; **(G)**: The expression level of SOD; **(H)** The expression level of AGEs; **(I)** The expression level of VIP; **(J)** The expression level of Ghrelin; ***: *p* < 0.001, **: *p* < 0.01, *: *p* < 0.05).

To verify the results of the network analysis, the regulation of inflammation and gastrointestinal motility by WDG was verified *in vivo*. The results showed that compared with the control group, the levels of TNF α (Figure 4E), IL-1β (Figure 4A), CRP (Figure 4C), IL-6 (Figure 4D), IL-10 (Figure 4B), MDA (Figure 4F), and SOD (Figure 4G) were significantly higher in the POI group, but there were lower in the WDG and Procapride groups, which showed that WDG decreased inflammation in POI rats, and oxidative stress level.

### 3.6 WDG improved gastrointestinal motility in POI rats

**FIGURE 5:**
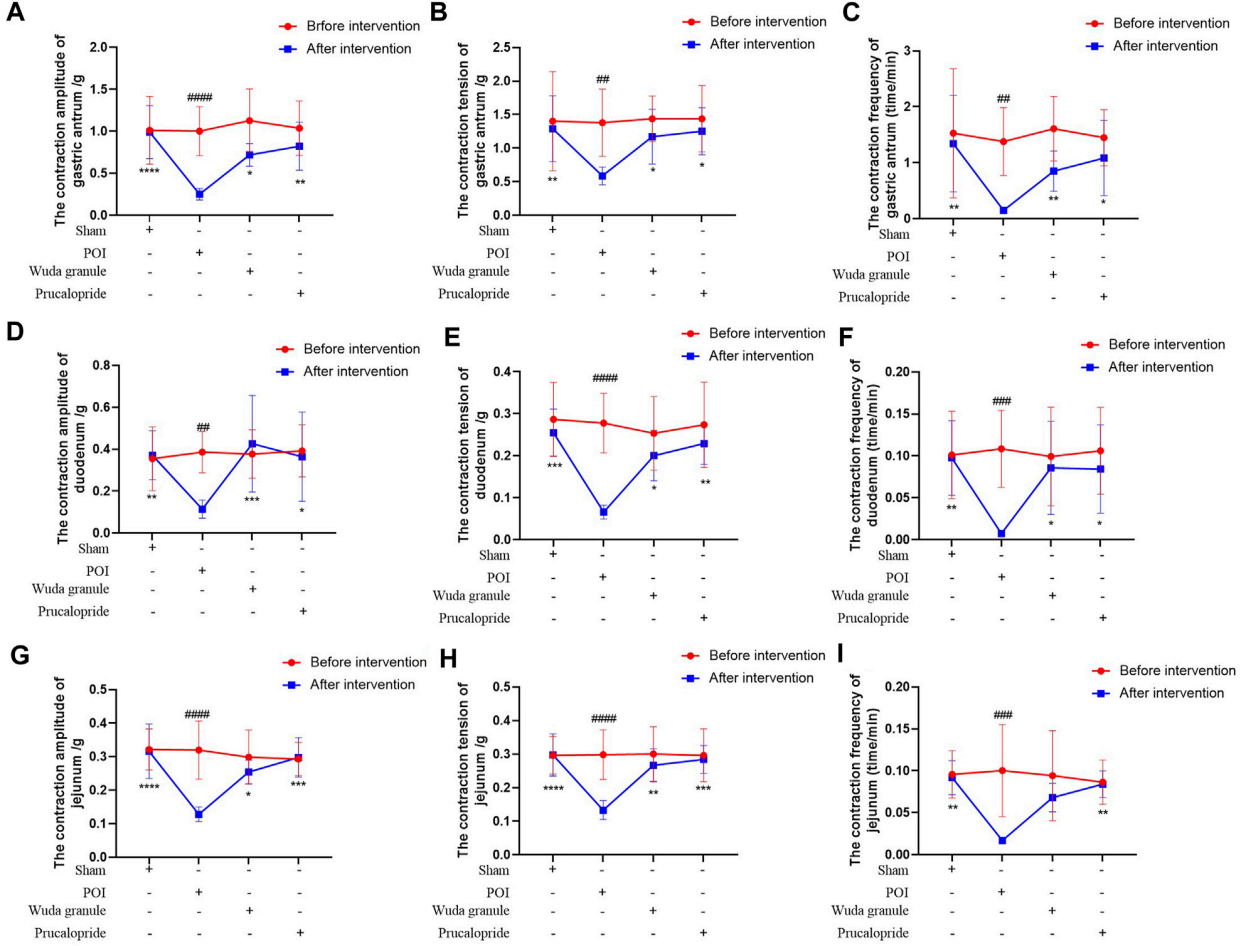
WDG improved the contraction amplitude, tension and frequency of gastric antrum, duodenum and jejunum in POI rats. (**(A)** The contraction amplitude of gastric antrum; **(B)** The contraction tension of gastric antrum; **(C)** The contraction frequency of gastric antrum; **(D)** The contraction amplitude of duodenum; **(E)** The contraction tension of duodenum; **(F)** The contraction frequency of duodenum; **(G)** The contraction amplitude of jejunum; **(H)** The contraction tension of jejunum; **(I)** The contraction frequency of jejunum; ***: Compared with 1 day after surgery; ###: Compared with before surgery; ∆∆∆ : Comparison between WDG group and placebo group).

Compared to that in the sham group, the expression of AGEs, VIP and ghrelin was lower in the POI group, but higher in the WDG and the Procapride groups (Figures 4H–J), indicating that symptoms of GMD could be alleviated by WDG, similar to the effect of Procapride, a drug proven for improving gastrointestinal motility in the clinic. These results were further verified by observing the contraction parameters of the gastric antrum, duodenum, and jejunum in the POI rat model. The results showed that the amplitude, tension, and frequency of contraction were reduced in the POI group after intervention, whereas in the WDG and Procapride groups, these were improved (Figures 5A–I), indicating that GMD could be successfully induced in the POI rats, and that WDG improved gastrointestinal motility.

### 3.7 Clinical characteristics of patients who had undergone laparoscopic colorectal cancer surgery

Among the 26 patients who had undergone laparoscopic colorectal cancer surgery, no significant difference in clinical characteristics was observed between the 12 patients of the placebo group and 14 of the WDG group (*p* > 0.05; Table 1). During the digestive process, MMC was the main form of gastrointestinal motility, it also had no significant difference between the two groups before treatment (*p* > 0.05; Table 1).

**TABLE 1:** The characteristics of included patients before intervention (n/%, M/P_25_-P_75_ or $\bar{x}\pm$ s).

Indicators	Placebo group (n = 12)	WDG group (n = 14)	$X^{2}$ /t/Z	*p*
Sex (male)	7/58.3%	5/35.7%	1.330	0.249
Age(y)	61.4 ± 8.1	56.9 ± 7.3	−1.501	0.146
Total flow time (d)	1(0.5–1.5)	1 (1–2)	0.930	0.352
Fasting time (h)	12 (12–12)	12 (12–24)	1.280	0.201
Anesthesia time (h)	4 (3.5–4)	4 (3–4)	−0.728	0.467
Operation time (h)	3.00 ± 0.74	2.79 ± 0.80	−0.704	0.488
Intraoperative flushing volume (mL)	1650 (1000–2100)	2000 (1600–2000)	1.196	0.232
Indwelling drainage tube time (d)	6 (5.5–7)	5.5(4–7)	−1.370	0.171
Indwelling catheter time (d)	3.17 ± 1.70	2.71 ± 1.38	−0.749	0.461
Number of MMC (per 2h)	1.33 ± 0.65	1.07 ± 0.62	−1.053	0.303
Time of phase I (min)	36.00 ± 46.01	23.71 ± 40.67	−0.723	0.477
Time of phase II (min)	46.83 ± 9.90	51.86 ± 11.51	1.182	0.249
Amplitude of phase II (mmHg)				
Antrum gastricum	45.98 ± 12.86	51.05 ± 11.87	1.044	0.307
Duodenum	25.58 ± 5.44	26.90 ± 5.76	0.596	0.557
Jejunum	24.31 ± 5.92	24.62 ± 4.39	0.155	0.878
Motility index of phase II (mmHg/min)				
Antrum gastricum	4828.92 ± 1286.39	3960.21 ± 1173.82	1.800	0.084
Duodenum	7204.67 ± 2721.35	7116.40 ± 2052.87	0.094	0.926
Jejunum	7411(6165.5–8902)	6177.5 (5644–7693)	−0.874	0.382

Note: Continuous variable data with normal distribution was presented as the mean ± standard deviation and was analyzed by Student’s t-tests, Continuous variable data with non-normal distribution was presented as M/P_25_-P_75_ and was analyzed by Mann–Whitney U tests. Categorical variable was presented as frequency/percentage (n/%) and was analyzed by Chi-squared test or fisher exact test. A value of *p* < 0.05 was considered statistically significant.

### 3.8 WDG improved gastrointestinal motility in patients

**FIGURE 6:**
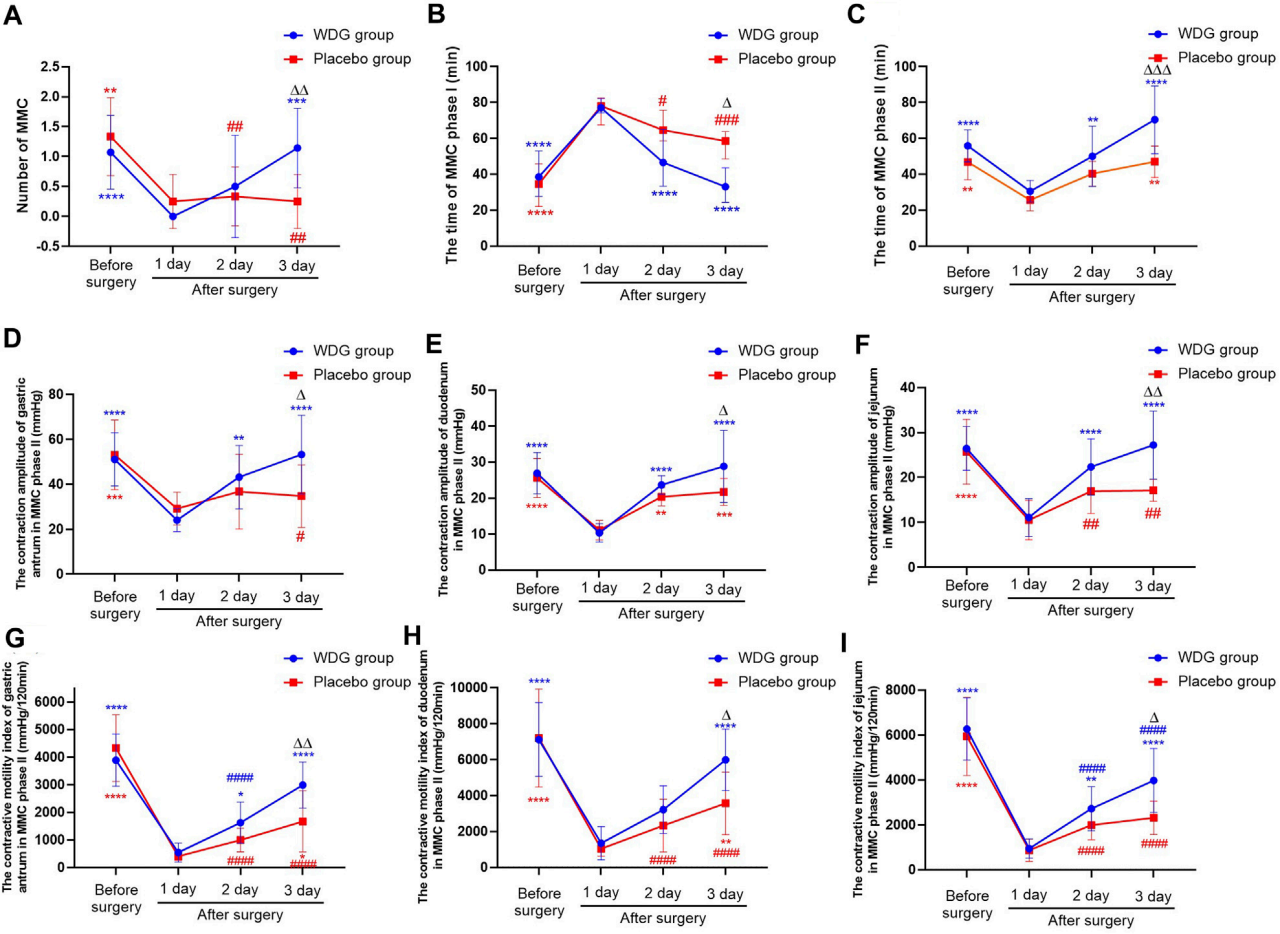
WDG improved gastrointestinal motility in patients. (**(A)** The numbers of MMC in the two groups; **(B)** The time of MMC phase I; **(C)** The time of MMC phase II; **(D)** The contraction amplitude of gastric antrum in MMC phase II (mmHg); **(E)** The contraction amplitude of duodenum in MMC phase II (mmHg); **(F)** The contraction amplitude of jejunum in MMC phase II (mmHg); **(G)** The contractive motility index of gastric antrum in MMC phase II (mmHg/120min); **(H)** The contractive motility index of duodenum in MMC phase II (mmHg/120min); **(I)** The contractive motility index of jejunum in MMC phase II (mmHg/120min); ***: Compared with 1 day after surgery; ###: Compared with before surgery; ∆∆∆ : Comparison between WDG group and placebo group).

The duration of the MMC phase I was significantly increased; further, 1 day after surgery, the duration of MMC phase II, the number of MMC, and the contractive amplitude and motility of the gastric antrum, duodenum, and jejunum, were decreased compared to the levels before surgery (*p* < 0.05; Figures 6A–I). However, compared with the placebo group, the above results were reversed with a time-dependent tendency for the WDG group, and on the third day after surgery, the gastrointestinal motility was significantly restored (*p* < 0.05), which indicated that WDG exerted a pharmacological effect on GMD by improving gastrointestinal motility.

### 3.9 WDG improved digestive function and reduced inflammation in patients

**FIGURE 7:**
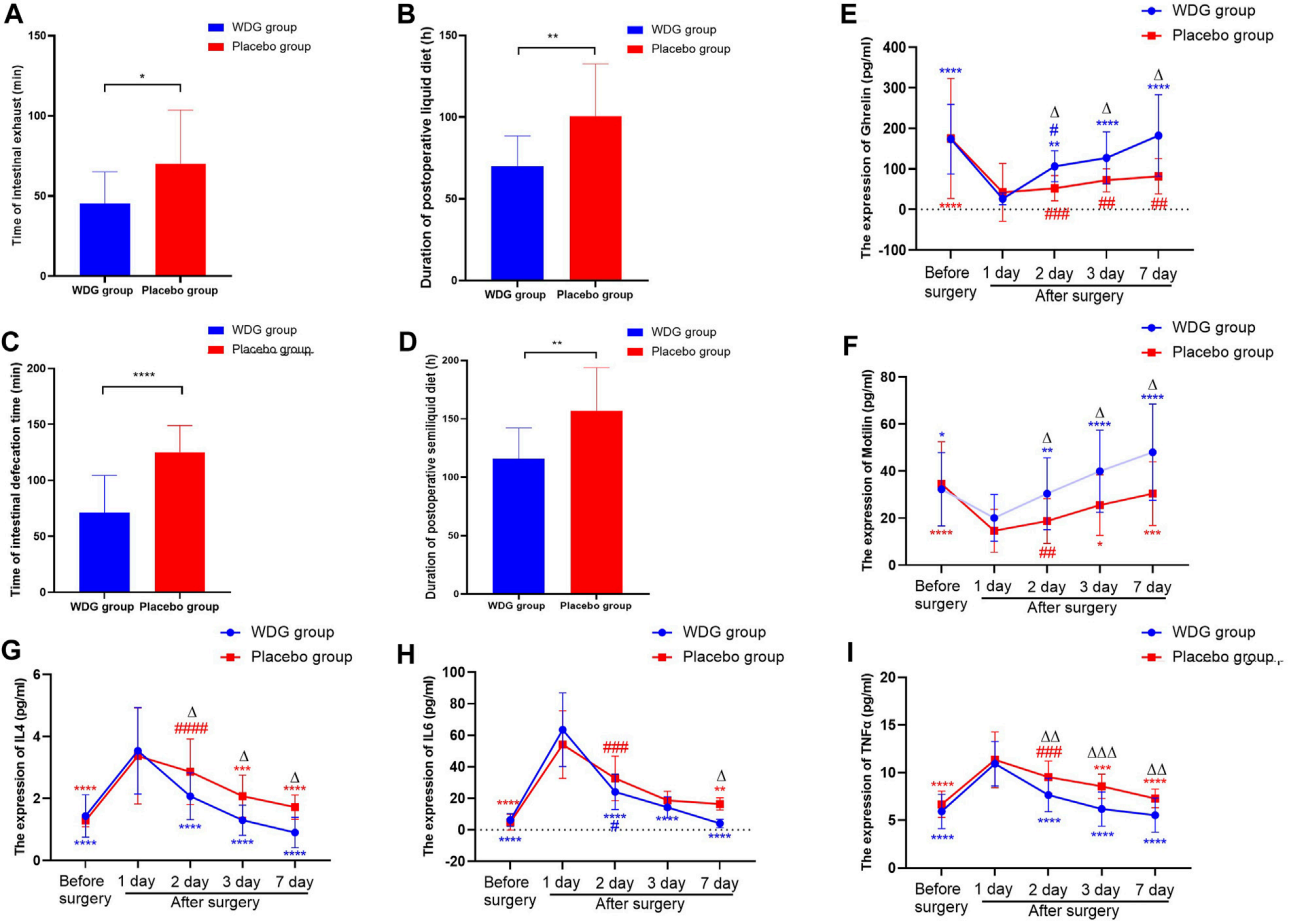
WDG improved gastrointestinal function and reduced inflammation in patients. (**(A)** The time of intestinal exhaust (min) in the two groups; **(B)** The time of intestinal defecation time (min) in the two groups; **(C)** The duration of postoperative liquid diet (h) after surgery in the two groups; **(D)** The duration of postoperative semiliquid diet (h) after surgery in the two groups; **(E)** The expression level of Ghrelin (pg/mL); **(F)** The expression level of Motilin (pg/mL); **(G)** The expression level of IL4 (pg/mL); **(H)** The expression of IL-6 (pg/mL); **(I)** The expression level of TNF-α (pg/mL); ***: Compared with 1 day after surgery; ###: Compared with before surgery; ∆∆∆ : Comparison between WDG group and placebo group).

The levels of IL4, IL6, and TNFα (Figures 7G–1) was significantly higher after surgery in patients, but were lower in the WDG group, especially on the third and seventh days after surgery. In addition, the levels of ghrelin and motilin were lower in the WDG group 1 day after surgery, which was further lowered with an increasing number of days (Figures 7E–F). Compared to the placebo administration, WDG treatment decreased the duration of postoperative intestinal exhaust and defecation, as well as the duration of postoperative liquid and semi-liquid diet recovery (Figures 7A–D). These results suggested that WDG exerts therapeutic effects on GMD by improving digestive function and reducing inflammation.

### 3.10 Screened key compounds of WDG could target core hub-targets of GMD

**FIGURE 8:**
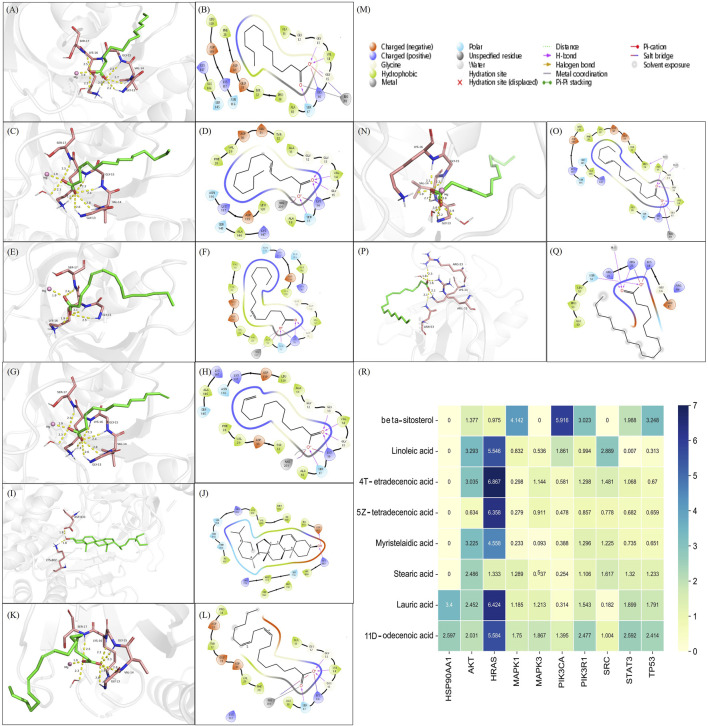
The active compounds of WDG had preferable docking ability with Hub-Target of GMD. **(A)** 3D docking structure of Lauric acid docked with HRAS; **(B)** 2D docking structure of Lauric acid docked with HRAS; **(C)** 3D docking structure of 4-Tetradecenoic acid docked with HRAS; **(D)** 2D docking structure of 4-Tetradecenoic acid docked with HRAS; **(E)** 3D docking structure of 5Z-tetradecenoic acid docked with HRAS; **(F)** 2D docking structure of 5Z-tetradecenoic acid docked with HRAS; **(G)** 3D docking structure of 11-Dodecenoic acid docked with HRAS; **(H)** 2D docking structure of 11-Dodecenoic acid docked with HRAS; **(I)** 3D docking structure of beta-sitosterol docked with PIK3CA; **(J)** 2D docking structure of beta-sitosterol docked with PIK3CA; **(K)** 3D docking structure of Linoleic acid docked with HRAS; **(L)** 2D docking structure of Linoleic acid docked with HRAS; **(M)** Type of interaction between identified compounds and their docking targets; **(N)** 3D docking structure of Myristelaidic acid docked with HRAS; **(O)** 2D docking structure of Myristelaidic acid docked with HRAS; **(P)** 3D docking structure of Stearic acid docked with AKT; **(Q)** 2D docking structure of Stearic acid docked with AKT; **(R)** Heat map of docking score of key WDG components docked with Hub-Target of GMD).

The prerequisite for efficacy of a drug is its ability to bind with specific proteins or receptors. Therefore, we verified whether the screened key compounds of WDG could bind with the hub targets of GMD. Network analysis identified beta-sitosterol, linoleic acid, 4-tetradecenoic acid, 5Z-tetradecenoic acid, myristelaidic acid, stearic acid, lauric acid and 11-dodecenoic acid as the key active components of WDG. Further, HSP90AA1, AKT, HRAS, MAPK1, MAPK3, PIK3CA, PIK3R1, SRC, STAT3, and TP53 were screened as the key targets. The results of molecular docking showed that linoleic acid, 4-tetradecenoic acid, 5Z-tetradecenoic acid, myristelaidic acid, lauric acid, and 11-dodecenoic acid had higher docking scores with HRAS; further, stearic acid and beta-sitosterol could dock with AKT and PIK3CAwith high docking scores respectively, indicating that these eight compounds of WDG may exert anti-GMD effects by targeting HRAS, AKT, and PIK3CA (Figures 8A–R). These results provided new insights for further research to explore the pharmacological compounds of WDG.

## 4 Discussion

Gastrointestinal motility disorder (GMD) is a common gastrointestinal disease that severely affects the patient’s life and physical wellbeing (Raubinger et al., 2022). It can cause incoordination of the gastrointestinal delay, delay gastric emptying, affect enteral feed intolerance, and even functionally obstruct the small or large intestines. GMD is a common public health issue worldwide and is the main complication or symptom of irritable bowel syndrome and other functional gastrointestinal disorders, particularly in patients undergoing gastrointestinal surgery (Wang et al., 2019). Presently, Western medical treatment cannot effectively target the pathogenesis of the disease because of its limited effect (Zhang and Bai, 2022). In this study, we aimed to explore the pharmacological mechanism of WDG in GMD via network analysis and experimental validation.

### 4.1 WDG alleviated GMD via an anti-inflammatory effect

Gastrointestinal symptoms, are significant non-motor symptoms of GMD, and are represented by inflammation (Zou et al., 2022), which is considered as an aggravating factor and manifestation of GMD (Villanacci et al., 2008). Gastrointestinal disorders promote inflammatory cell infiltration of the mucosa and/or myenteric plexus (Bassotti et al., 2009), which leads to abnormalities in the enteric nervous system, and alterations in normal motor function and visceral reflexes of the gut (Bassotti et al., 2014). Inflammation and GMD have been proposed as factors confounding with each other (Peuhkuri et al., 2010). Various studies have shown that the release of inflammatory cytokines accelerates the development of gastrointestinal disorders (Docsa et al., 2022), which can activate resident macrophages and T helper cells and recruit neutrophils into the smooth muscle to propagate the spread of motility dysfunction along the gastrointestinal tract (Wehner et al., 2007). Thus, specific or combined inflammatory mediators such as C-X-C motif chemokine ligand 1 (also known as GroA and upregulated in developed ileus), are believed to be viable biomarkers for the early detection of gastrointestinal disorders (Docsa et al., 2020). In this study, we predicted that inflammation may be the main biological process in GMD that is targeted by WDG. Experiments using the POI rat model showed that WDG did decrease the expression of inflammatory markers IL-10, IL-6, IL-1β, CRP, and TNF-α. Further, the clinical experiment revealed that treatment with WDG could also decrease the levels of IL-6, IL4, and TNF-α. These results proved that WDG exerts an anti-inflammatory effect in GMD, thus elucidating one of the mechanisms for the pharmacological effect of WDG on GMD.

### 4.2 WDG alleviated GMD by improving gastrointestinal motility

Gastrointestinal motility disorder is considered an idiopathic symptom of other diseases and is characterized by abnormal gastrointestinal contractions, dysphagia, gastroesophageal reflux disease, flatulence, severe constipation, vomiting, and abdominal distension (Fukuda et al., 2022). In GMD, gastrointestinal motility is impaired mainly because of problems within the muscles that control peristalsis or with the nerves or hormones that govern muscle contractions. It has been reported that prokinetic motility drugs can alleviate the symptoms of patients with GMD by promoting gastric emptying and accommodation rates (Shrestha et al., 2021). Commonly, gastrointestinal motility drugs have certain curative effects; however, all of these cause different degrees of side effects and do not effectively reduce the recurrence rate following drug withdrawal (Wang et al., 2022). Traditional Chinese medicines are believed to play an important role in regulating gastrointestinal motility and alleviating GMD symptoms (Zhang et al., 2022). These medicines can increase the function of muscles, nerves, and hormones by regulating excitatory neurotransmission (Chen et al., 2022), gut microbiota (Yang et al., 2022), intestinal glial cell apoptosis (Wang et al., 2022), and nitrergic neurons (Li et al., 2022). Thus, these medicines can potentially improve GMD by enhancing the motility of muscle contraction. In this study, treatment with WDG increased the levels of motilin, AGEs, VIP, and ghrelin, which are the indicators for the assessment of gastrointestinal motility. In addition, WDG treatment improved the contraction of the gastric antrum, duodenum, and jejunum in the POI rat model, decreased the duration of the MMCI phase, and increased the duration of MMC phase II, the number of MMC, and the contractive amplitude and motility index of the gastric antrum, duodenum, and jejunum. Interestingly, WDG treatment also reduced the duration of postoperative intestinal exhaust and defecation, as well as the duration of postoperative liquid and semi-liquid diet recovery.

### 4.3 WDG alleviates GMD via multi-compounds, multi-targets, and multi-pathways

Chinese herbal medicines, which consist of natural medicinal herbs, possess various complex chemical compositions owing to the herbal combinations, making it difficult to elucidate the mechanism of their therapeutic effects. A single herb or Chinese medicinal formula contains many phytochemical compounds, including alkaloids, terpenoids, and flavonoids. These medicines achieve the desired pharmacological effects mainly through a combination of various compounds. Many effective drugs in the clinic have been derived from Chinese herbal medicines, such as artemisinin (named qinghaosu in China, derived from Artemisia annua L. [Asteraceae]), which not only increase the pharmacological effect, but also reduce the cost for patients and make the pharmacokinetics clearer. Thus, identifying the main chemical constituents responsible for the therapeutic function of Chinese herbal medicine is vital. In this study, we predicted eight key active compounds in WDG that may be responsible for its therapeutic effects. Previous studies have reported that beta-sitosterol (Kasirzadeh et al., 2021), stearic acid (Sobocinska et al., 2022), linoleic acid (Zhang et al., 2021), and lauric acid (Li et al., 2022) exert pharmacological effects on the regulation of intestinal inflammation and the intestinal gut function, both of which are consistent with the mechanism of GMD. However, there remains a lack of reports on the effect of 4-tetradecenoic acid, 5Z-tetradecenoic acid, myristelaidic acid, and 11-dodecenoic acid on gastrointestinal disorders, prompting us to focus on these drugs with potential functions in GMD in future studies. In addition, results of the molecular docking analysis suggested that these eight key compounds target different proteins, thus suggesting that these compounds may be responsible for the therapeutic effects of WDG on GMD mainly by targeting multitargets.

### 4.4 Implications on prospective research and limitations of present study

This study aimed to explore the potential pharmacological effects of WDG on GMD, and their underlying mechanisms, providing insight for the research on the mechanism of WDG on GMD. It firstly explored the mechanism of action of WDG in treating postoperative GMD through network analysis, animal experiments, and small sample clinical controlled studies. The results showed that WDG could promote the recovery of postoperative gastrointestinal motility via inhibiting the inflammatory process and promoting postoperative gastrointestinal motility recovery, providing the biochemical basis of WDG’s clinical efficacy. Although the results were encouraging, there still exist some limitations in this study. As a preliminary exploration, the relationship and crosstalk between the inflammatory mechanism of Wuda granules and the promoting gastrointestinal motility effect still lack further verification, which should be verified in the future research. Network analysis was mainly depended on compute calculation, it may lead to a very speculative hypothesis, which may result in false positives, further verification on the compounds of herbs should be conducted to confirm the results. Thus, in future, the research should be designed according to the following directions: 1) the pathway involving in inhibiting inflammation and promoting gastrointestinal motility by WDG should be designed to further verify the potential pathway of WDG based on the results of network analysis. 2) In clinic, high-quality RCT with large sample and multiple center should be designed to assess the anti-inflammation effect and effect of promoting gastrointestinal motility based on the small sample results of this study. 3) with multitopic analysis and sequencing techniques, the mechanism involving in the therapeutical effect of WDG should be confirmed with human sample. 4) as various effective compounds has been derived from Chinese herds medicine in recent years, the eight key compounds screened in this study should be further explored for their role in the alleviation of GMD with the purpose of developing plant-derived compounds that may have better therapeutic effects.

## 5 Conclusion

This study firstly demonstrated that treatment with WDG alleviates GMD mainly by reducing inflammation and promoting gastrointestinal motility, multi-compounds and multi-targets are involved in this process, providing new insights to understand the effect of WDG treatment on GMD, which can inspire future research, and serve as a reference to devise clinical strategies in the treatment of GMD.

## Data Availability

The datasets presented in this study can be found in online repositories. The names of the repository/repositories and accession number(s) can be found in the article/Supplementary Material.

## Abbreviations

DL, Drug-likeness; GMD, Gastrointestinal motility disorders; GO, Gene ontology; HCT, Herbal-compounds-targets; KEGG, Kyoto Encyclopedia of Gene and Genome; MI, Motor index; MMC, Migrating motor complex; OB, Oral bioavailability; POI, Postoperative ileus; PPI, Protein-protein interaction; TCM, Traditional Chinese medicine; WDG, Wuda granule.

## References

[B1] Accarie A. Vanuytsel T. (2020). Animal models for functional gastrointestinal disorders. Front. PSYCHIATRY 11, 509681. 10.3389/fpsyt.2020.509681 33262709 PMC7685985

[B2] Bassotti G. Antonelli E. Villanacci V. Salemme M. Coppola M. Annese V. (2014). Gastrointestinal motility disorders in inflammatory bowel diseases. World J. Gastroenterol. 20, 37–44. 10.3748/wjg.v20.i1.37 24415856 PMC3886030

[B3] Bassotti G. Villanacci V. Nascimbeni R. Cadei M. Fisogni S. Antonelli E. (2009). Enteric neuroglial apoptosis in inflammatory bowel diseases. J. CROHNS COLITIS 3, 264–270. 10.1016/j.crohns.2009.06.004 21172285

[B4] Chen W. Liao L. Huang Z. Lu Y. Lin Y. Pei Y. (2022). Patchouli alcohol improved diarrhea-predominant irritable bowel syndrome by regulating excitatory neurotransmission in the myenteric plexus of rats. Front. Pharmacol. 13, 943119. 10.3389/fphar.2022.943119 36452228 PMC9703083

[B5] Docsa T. Bhattarai D. Sipos A. Wade C. E. Cox C. J. Uray K. (2020). CXCL1 is upregulated during the development of ileus resulting in decreased intestinal contractile activity. Neurogastroenterol. Motil. 32, e13757. 10.1111/nmo.13757 31722447

[B6] Docsa T. Sipos A. Cox C. S. Uray K. (2022). The role of inflammatory mediators in the development of gastrointestinal motility disorders. Int. J. Mol. Sci. 23, 6917. 10.3390/ijms23136917 35805922 PMC9266627

[B7] Foong D. Zhou J. Zarrouk A. Ho V. O'Connor M. D. (2020). Understanding the biology of human interstitial cells of cajal in gastrointestinal motility. Int. J. Mol. Sci. 21, 4540. 10.3390/ijms21124540 32630607 PMC7352366

[B8] Fukuda H. Sato H. Fujiyoshi Y. Abe H. Okada H. Shiota J. (2022). Risks of refractory chest pain after peroral endoscopic myotomy in achalasia-related esophageal motility disorders: Short-term results from a multicenter study in Japan. Gastrointest. Endosc. 96, 620–629.e4. 10.1016/j.gie.2022.04.1347 35568241

[B9] Jiang Z. Cao L. X. Liu B. Chen Q. C. Shang W. F. Zhou L. (2017). Effects of Chinese herbal medicine Xiangbin prescription on gastrointestinal motility. World J. Gastroenterol. 23, 2987–2994. 10.3748/wjg.v23.i16.2987 28522917 PMC5413794

[B10] Jiang Z. Chen Q. Zhang J. Cao L. Chen Z. (2020). Wuda granule, a traditional Chinese herbal medicine, ameliorates postoperative ileus by anti-inflammatory action. Pathol. Res. Pract. 216, 152605. 10.1016/j.prp.2019.152605 31974003

[B11] Kalff J. C. Schraut W. H. Simmons R. L. Bauer A. J. (1998). Surgical manipulation of the gut elicits an intestinal muscularis inflammatory response resulting in postsurgical ileus. Ann. Surg. 228, 652–663. 10.1097/00000658-199811000-00004 9833803 PMC1191570

[B12] Kasirzadeh S. Ghahremani M. H. Setayesh N. Jeivad F. Shadboorestan A. Taheri A. (2021). *β*-Sitosterol alters the inflammatory response in CLP rat model of sepsis by modulation of NF*κ*B signaling. Biomed. Res. Int. 2021, 5535562. 10.1155/2021/5535562 33997001 PMC8105092

[B13] Lai X. Wang X. Hu Y. Su S. Li W. Li S. (2020). Editorial: Network pharmacology and traditional medicine. Front. Pharmacol. 11, 1194. 10.3389/fphar.2020.01194 32848794 PMC7417929

[B14] Li S. Zhang B. (2013). Traditional Chinese medicine network pharmacology: Theory, methodology and application. Chin. J. Nat. Med. 11, 110–120. 10.1016/S1875-5364(13)60037-0 23787177

[B15] Li X. Yi Y. Wu J. Yang Q. Tan B. Chi S. (2022a). Identification of a chromosome 1 substitution line B6-Chr1BLD as a novel hyperlipidemia model *via* phenotyping screening. Metabolites 12, 1276. 10.3390/metabo12121276 36557314 PMC9781061

[B16] Li Y. R. Li Y. Jin Y. Xu M. Fan H. W. Zhang Q. (2022b). Involvement of nitrergic neurons in colonic motility in a rat model of ulcerative colitis. World J. Gastroenterol. 28, 3854–3868. 10.3748/wjg.v28.i29.3854 36157548 PMC9367233

[B17] Madl C. Madl U. (2018). Gastrointestinal motility in critically ill patients. Med. Klin. Intensivmed. Notfmed 113, 433–442. 10.1007/s00063-018-0446-6 29802424

[B18] Patil K. R. Mahajan U. B. Unger B. S. Goyal S. N. Belemkar S. Surana S. J. (2019). Animal models of inflammation for screening of anti-inflammatory drugs: Implications for the discovery and development of phytopharmaceuticals. Int. J. Mol. Sci. 20, 4367. 10.3390/ijms20184367 31491986 PMC6770891

[B19] Peuhkuri K. Vapaatalo H. Korpela R. (2010). Even low-grade inflammation impacts on small intestinal function. World J. Gastroenterol. 16, 1057–1062. 10.3748/wjg.v16.i9.1057 20205274 PMC2835780

[B20] Raubinger S. Allworth S. Carey S. (2022). When you are living and dying at the same time" - a qualitative exploration of living with gastrointestinal motility disorders. J. Hum. Nutr. Diet. 36, 622–631. 10.1111/jhn.13114 36420640

[B21] Shrestha D. B. Budhathoki P. Subedi P. Khadka M. Karki P. Sedhai Y. R. (2021). Acotiamide and functional dyspepsia: A systematic review and meta-analysis. Cureus 13, e20532. 10.7759/cureus.20532 35070565 PMC8765587

[B22] Singh R. Zogg H. Ghoshal U. C. Ro S. (2022). Current treatment options and therapeutic insights for gastrointestinal dysmotility and functional gastrointestinal disorders. Front. Pharmacol. 13, 808195. 10.3389/fphar.2022.808195 35145413 PMC8822166

[B23] Sobocinska M. Fichna J. Gieldon A. Skowron P. Kamysz E. (2022). N-terminally lipidated sialorphin analogs-synthesis, molecular modeling, *in vitro* effect on enkephalins degradation by NEP and treatment of intestinal inflammation in mice. Int. J. Mol. Sci. 23, 14450. 10.3390/ijms232214450 36430928 PMC9695599

[B24] Suresh H. Zhou J. Ho V. (2021). The short-term effects and tolerability of low-viscosity soluble fibre on gastroparesis patients: A pilot clinical intervention study. NUTRIENTS 13, 4298. 10.3390/nu13124298 34959850 PMC8704257

[B25] Villanacci V. Bassotti G. Nascimbeni R. Antonelli E. Cadei M. Fisogni S. (2008). Enteric nervous system abnormalities in inflammatory bowel diseases. Neurogastroenterol. Motil. 20, 1009–1016. 10.1111/j.1365-2982.2008.01146.x 18492026

[B26] Wang S. Yan M. Guo Y. Sun R. Jin H. Gong Y. (2020a). *In vivo* and *in vitro* effects of Salsola collina on gastrointestinal motility in rats. Iran. J. BASIC Med. Sci. 23, 383–389. 10.22038/IJBMS.2019.40613.9605 32440326 PMC7229513

[B27] Wang T. Xu Y. Chen Q. Zheng W. Wang J. Zeng H. (2020b). Metabolomics analysis of laparoscopic surgery combined with Wuda granule to promote rapid recovery of patients with colorectal cancer using UPLC/Q-TOF-MS/MS. Evid. Based Complement. Altern. Med. 2020, 5068268. 10.1155/2020/5068268 PMC7040410 32104193

[B28] Wang X. Liu H. Li W. Xiao H. (2022a). Bibliometric analysis of functional dyspepsia research trends over the past 20 years. Front. Public Health 10, 1019110. 10.3389/fpubh.2022.1019110 36504925 PMC9727411

[B29] Wang X. M. Lv L. X. Qin Y. S. Zhang Y. Z. Yang N. Wu S. (2022b). Ji-Chuan decoction ameliorates slow transit constipation via regulation of intestinal glial cell apoptosis. World J. Gastroenterol. 28, 5007–5022. 10.3748/wjg.v28.i34.5007 36160643 PMC9494937

[B30] Wang X. Wang Z. Y. Zheng J. H. Li S. (2021). TCM network pharmacology: A new trend towards combining computational, experimental and clinical approaches. Chin. J. Nat. Med. 19, 1–11. 10.1016/S1875-5364(21)60001-8 33516447

[B31] Wang X. Zhang C. Zheng M. Gao F. Zhang J. Liu F. (2019). Metabolomics analysis of L-arginine induced gastrointestinal motility disorder in rats using UPLC-MS after magnolol treatment. Front. Pharmacol. 10, 183. 10.3389/fphar.2019.00183 30881305 PMC6405429

[B32] Wehner S. Behrendt F. F. Lyutenski B. N. Lysson M. Bauer A. J. Hirner A. (2007). Inhibition of macrophage function prevents intestinal inflammation and postoperative ileus in rodents. GUT 56, 176–185. 10.1136/gut.2005.089615 16809419 PMC1856749

[B33] Yang L. Wang Y. Zhang Y. Li W. Jiang S. Qian D. (2022). Gut microbiota: A new avenue to reveal pathological mechanisms of constipation. Appl. Microbiol. Biotechnol. 106, 6899–6913. 10.1007/s00253-022-12197-2 36190540

[B34] Yang M. Chen J. L. Xu L. W. Ji G. (2013). Navigating traditional Chinese medicine network pharmacology and computational tools. Evid. Based Complement. Altern. Med. 2013, 731969. 10.1155/2013/731969 PMC3747450 23983798

[B35] Zeng H. Wang W. Cao L. Wu Y. Ouyang W. Diao D. (2022). Effect of Wuda granule on gastrointestinal function recovery after laparoscopic intestinal resection: A randomized-controlled trial. Gastroenterol. Rep. (Oxf) 10, goac004. 10.1093/gastro/goac004 35186297 PMC8849281

[B36] Zhang J. Wang X. Wang F. Tang X. (2022). Xiangsha Liujunzi Decoction improves gastrointestinal motility in functional dyspepsia with spleen deficiency syndrome by restoring mitochondrial quality control homeostasis. PHYTOMEDICINE 105, 154374. 10.1016/j.phymed.2022.154374 35963194

[B37] Zhang Q. Zhong D. Ren Y. Y. Meng Z. K. Pegg R. B. Zhong G. (2021). Effect of konjac glucomannan on metabolites in the stomach, small intestine and large intestine of constipated mice and prediction of the KEGG pathway. FOOD Funct. 12, 3044–3056. 10.1039/d0fo02682d 33710209

[B38] Zhang X. P. Bai X. H. (2022). Analysis on the gastrointestinal motility disorder of gastroesophageal reflux disease and the mechanism of acupuncture-moxibustion from the perspective of autonomic nervous system. Zhongguo Zhen Jiu 42, 1299–1303. 10.13703/j.0255-2930.20220608-k0007 36397230

[B39] Zou Y. Ye F. Kong Y. Hu X. Deng X. Xie J. (2022). The single-cell landscape of intratumoral heterogeneity and the immunosuppressive microenvironment in liver and brain metastases of breast cancer. Adv. Sci. (Weinh) 10, e2203699. 10.1002/advs.202203699 36529697 PMC9929130

