# Supplementary figures and images for: Therapeutic role of Wuda granule in gastrointestinal motility disorder through promoting gastrointestinal motility and decreasing inflammatory level
Source: Front Pharmacol. 2023 Aug 21;14:1237686. doi: 10.3389/fphar.2023.1237686 (PMC10476622; doi:10.3389/fphar.2023.1237686)

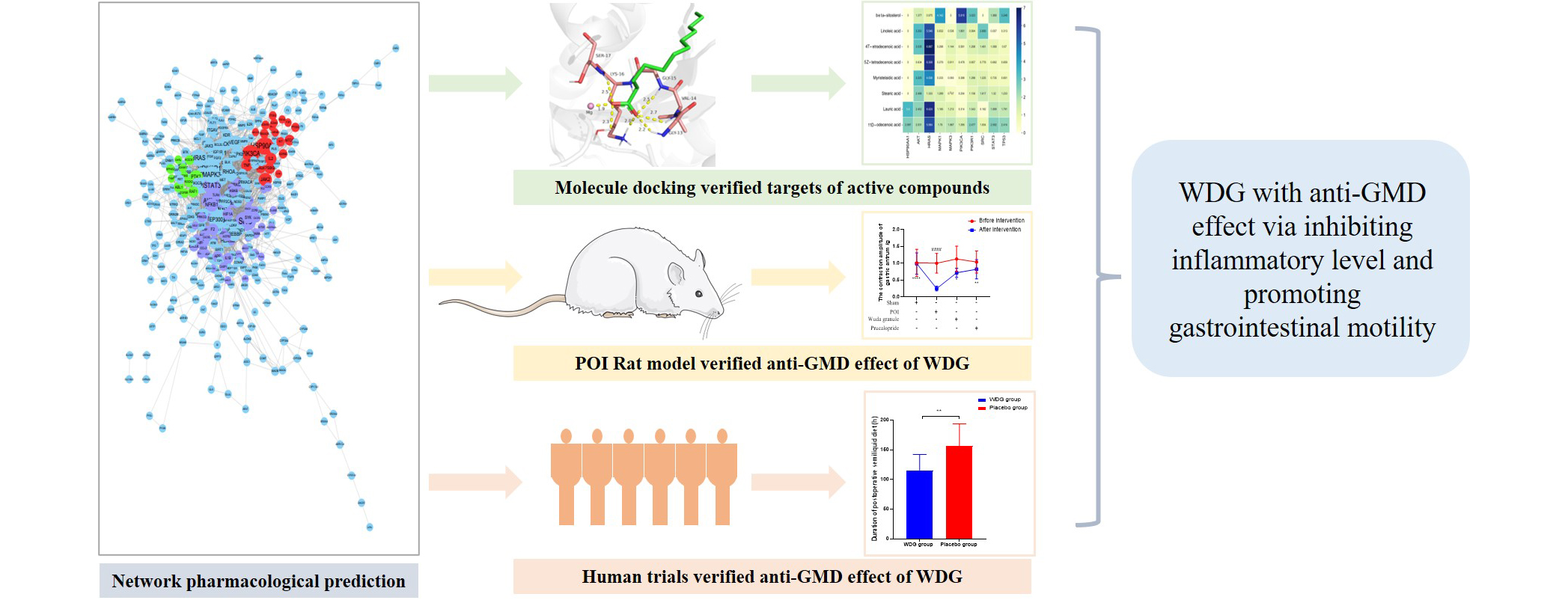

Supplement: Supplementary file 1 [file Image1.JPEG]
